# Stem cells, inflammation and allergy

**DOI:** 10.1186/1710-1492-5-13

**Published:** 2009-12-07

**Authors:** Marie-Renee Blanchet, Kelly M McNagny

**Affiliations:** 1 The Biomedical Research Centre, 2222 Health Sciences Mall, University of British Columbia, Vancouver, British Columbia, V6T 1Z3, Canada

## Abstract

Recently, many studies have suggested a potential role for early hematopoietic progenitor cell and hematopoietic stem cell (HSC) recruitment and differentiation in the development of allergy and inflammation. This is based largely on evidence that stem cells or CD34+ progenitor cells are recruited to the site of inflammation in allergic diseases, likely through many of the same adhesion and chemokine receptors used for stem cell homing to the bone marrow (PSGL-1, CXCL12, alpha4-beta1 integrin, CD44, etc). Once at the site of inflammation, it has been suggested that stem cells could participate in the perpetuation of inflammation by maturing, locally, into inflammatory cells in response to the growth factors released in situ. Here we provide a brief review of the evidence to suggest that hematopoietic stem and progenitor cells (versus mature hematopoietic lineages) are, indeed, recruited to the site of allergic inflammation. We also discuss the molecules that likely play a role in this process, and highlight a number of our novel observations on a specific role for the stem cell antigen CD34 in this process.

## Introduction

Recently, many studies have suggested a potential role for early hematopoietic progenitor cell and hematopoietic stem cell (HSC) recruitment and differentiation in the development of allergy and inflammation. This is based largely on evidence that stem cells or CD34+ progenitor cells are recruited to the site of inflammation in allergic diseases, likely through many of the same adhesion and chemokine receptors used for stem cell homing to the bone marrow (PSGL-1, CXCL12, α4β1 integrin, CD44, etc). Once at the site of inflammation, it has been suggested that stem cells could participate in the perpetuation of inflammation by maturing, locally, into inflammatory cells in response to the growth factors released *in situ*. This is further supported by the recent observation that transplantable HSCs, with the ability to reconstitute all hematopoietic lineages in irradiated hosts, can readily be isolated from the thoracic duct lymph of mice.

Many of these studies have relied on the use of the CD34 marker to identify HSCs. Antibodies to CD34 have been extremely useful in stem cell purification for clinical use and in furthering the understanding of stem cell biology. Interestingly, through more in-depth analyses, the known distribution of CD34 *in vivo *has recently expanded to include many of the key cell types that participate in allergic inflammation including mast cell and dendritic cell precursors, and eosinophils [1-4]. Careful analysis of these cell types derived from CD34-deficient mice has led to a better understanding of the exact functional role of CD34 in both stem cell migration and mucosal inflammatory cell homing [4,5]. Here we provide a brief review of the evidence to suggest that hematopoietic stem and progenitor cells (versus mature hematopoietic lineages) are, indeed, recruited to the site of allergic inflammation. We also discuss the molecules that likely play a role in this process, and highlight a number of our novel observations on a specific role for the stem cell antigen CD34 in this process.

### Hematopoietic stem cells and precursors respond to inflammatory stimuli both in bone marrow and in peripheral tissues

#### Recruitment of hematopoietic precursors in the lung

There has been a steadily increasing body of literature to suggest that inflammatory stimuli can have a potent effect on hematopoietic precursors in the bone marrow as well as recruitment of these cells to the site of inflammation, particularly in the lung [6]. Indeed, Denburg and colleagues have shown that eosinophil precursors are elevated in the bone marrow during development of upper and lower airway inflammation, and could contribute to the continuous production of eosinophils in asthma. Using a mouse model of upper (allergic rhinitis) and lower (asthma) airway inflammation, this study showed that at 24 h post challenge, the number of eosinophil progenitors is increased in the bone marrow after either upper airway challenge, lower airway challenge, or upper and lower airway challenges with ovalbumin. This increase correlated with a boost in mature eosinophils in the blood and tissue, with a spike in the production of IL-5 and eotaxin. It was further suggested that this increase in eosinophil progenitors could contribute to the well-described augmentation in mature eosinophil numbers in blood and tissue in these models.

### Inflammation and recruitment of eosinophils progenitors

**Figure 1:**
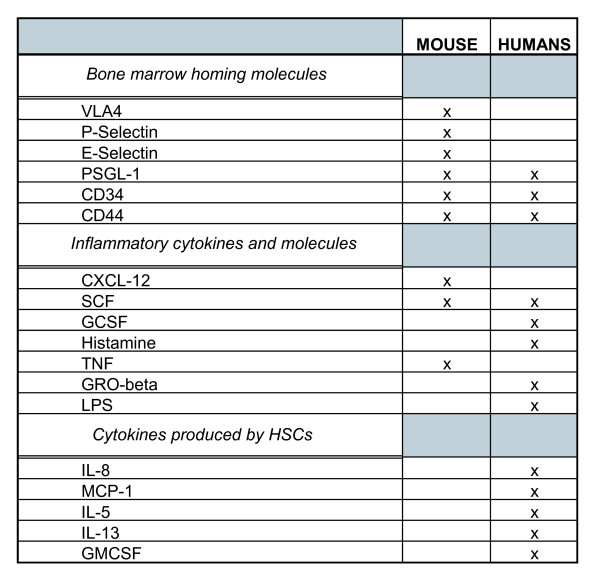
**Mouse and human expression of shared molecules between inflammation and stem cells**. Comparison of mouse and human expression of 1) bone marrow homing molecules influencing inflammation, 2) inflammatory molecules influencing bone marrow trafficking and 3) inflammatory cytokines produced by hematopoietic stem cells.

In addition to this evidence several additional studies, conducted in both humans and mice, support the hypothesis that cytokines and chemokines produced during inflammation influence recruitment and trafficking of eosinophil progenitors (see figure 1 for a complete list of molecules and receptors in human and mouse hematopoietic stem cells). For example, it was shown that in humans, inhaled IL-5 provokes a decrease in CD34+/CCR3 (eotaxin receptor)+ cells in bone marrow aspirates and bronchial mucosa, likely due to an increase in recruitment of these cells to the airway lumen [7]. Allergen challenge has also been shown to provoke an increase in CD34+/IL5R+ cells in the human airway lumen [8], as well as an increase in CCR3 expression by CD34+ and CD34+/IL-5+ populations in bone marrow aspirates [9]. Finally, this CD34+/CCR3+ progenitor population was shown to migrate *in vitro *towards the CCR3 ligand, eotaxin. The idea that cytokines, chemokines or antigen challenge can provoke the production and migration of eosinophil progenitors is very interesting indeed, since it supports the hypothesis that hematopoietic progenitor production in bone marrow and peripheral migration can be influenced by an inflammatory environment at distal sites, and that a constant production of hematopoietic progenitors promoted by inflammatory mediators could contribute to the chronicity of inflammatory diseases.

### Key hematopoietic stem cell homing receptors are used for inflammatory cell migration

#### Bone marrow homing receptors are also essential in inflammation

With a detailed evaluation of the molecules involved in hematopoietic cell migration and homing to the bone marrow, it quickly becomes apparent that these same receptors are essential for the efficient homing of mature effector cells to the site of inflammation. For example, the α4β1 integrin, VLA-4, is a key adhesive receptor for HSCs in the bone marrow and systemic and administration of VLA-4 blocking antibodies increases the number of hematopoietic progenitor cells in circulation[10,11]. Similarly, the inflammatory homing selectin ligand, PSGL-1 and its endothelial cell receptors P- and E-selectin, are also known to play a key role in homing and adhesion of HSCs to their bone marrow niche [12]. Interestingly, activation of PSGL-1 by a soluble ligand or an anti-PSGL-1 antibody leads to suppression of hematopoietic progenitor cell proliferation [13]. The hyaluronic acid (HA) receptor, CD44, is also known to play a role in both HSC homing and adhesion to their bone marrow niche [14] and to enhance the adhesion of inflammatory cells to endothelium at the sites of inflammation [15,16]. Of further interest, CD44 ligation is also known to stimulate eosinophil precursor proliferation [17] and could thereby enhance inflammatory cell expansion. Finally, we have shown that CD34, itself, is expressed by both HSCs and a number of inflammatory cell subsets (eosinophils, mast cells, dendritic cell precursors, etc). Interestingly, on both populations, CD34 appears to act as a type of molecular "Teflon" to enhance cell mobility and invasiveness and thereby facilitate trafficking of HSCs to the bone marrow and inflammatory cells to inflamed peripheral tissues [2,4,5]. In summary, the same repertoire of functional adhesion, homing and trafficking receptors are expressed by HSCs and inflammatory cells and should endow these cells with a similar potential for migration.

#### Inflammatory cytokines and chemo attractants are essential for bone marrow trafficking

Intriguingly, just as HSCs and inflammatory cells appear to use the same slate of adhesion and homing receptors for migration to the bone marrow and sites of inflammation, they also rely on the same chemo attractants, growth factors and cytokines. For example, CXCR4/CXCL12, a general inflammatory chemokine receptor and chemokine ligand, is the only known chemokine receptor expressed by HSCs and plays an instrumental role in HSC homing and retention in the bone marrow niche [18,19]. Likewise, c-kit, the receptor for stem cell factor (SCF) and a known regulator of stem cell growth and proliferation is also expressed by peripheral tissue mast cells and regulates their chemotaxis, survival, and expansion [20,21]. Moreover, G-CSF (a general inflammatory mediator) leads to neutrophil precursor activation in the bone marrow, MMP release, and proteolytic cleavage of c-kit, CXCL12 and CXCR4, etc. Cleavage of these molecules effectively deletes the molecular anchors that attract and hold HSCs in the bone marrow and leads to their rapid release into peripheral blood. Further, this blinds HSCs to signals that could recruit them back to the bone marrow until *de novo *CXCR4 and c-kit membrane receptors can be expressed on the HSC surface [22,23].

#### Inflammatory mediators and stem cell recruitment to the site of inflammation

In addition to facilitating the potent recruitment of peripheral blood cells to the site of local inflammation, there is accumulating evidence to suggest that inflammatory mediators can influence stem cell recruitment and migration as well. For example, mast cell precursors respond to CXCL12 and migration of the precursors is enhanced by the presence of histamine (signalling through the H4 receptors on mast cell precursors) or supernatants from IgE-stimulated mast cell cultures [24]. It has also been suggested that the migration of stem cells to chemotactic ligands is enhanced by the presence of TNF-alpha [25]. Another study suggests that the CXCR2 ligand GRO-beta mobilizes early stem cells [26]. These observations, combined with the array of inflammatory homing receptors expressed on this population suggest that recruitment of stem cells and hematopoietic progenitors may occur in allergy and chronic inflammation. If they were to differentiate into mature inflammatory cells *in situ*, these cells would be well positioned to influence the chronicity of disease. Since HSC are known to exit and re-enter the bone marrow through the circulation on a regular basis [27], they could easily be recruited to the site of inflammation during their normal homeostatic migration.

Recently, a study by Massberg et al suggested that HSC are, indeed, recruited from the bone marrow into the circulation and travel to the liver, peripheral blood, lung, small intestine and kidney. These authors also detected HSCs in thoracic duct lymph, which suggests that they have traveled through the lymph nodes and exited via the efferent lymphatics [28]. In this context, it is intriguing to speculate on the ability of HSCs to respond to inflammatory signals by accelerated differentiation into effector cells *in situ*. Support for this notion was recently provided by the observation that HSCs express the innate pathogen pattern recognition receptor, TLR4, and in response to LPS (a TLR4 ligand), these cells proliferate vigorously *in situ*, and differentiate into mature hematopoietic lineages (dendritic cells) [29]. This is the first evidence to suggest that recruitment, proliferation and accelerated maturation of circulating HSCs in response to an allergen or bacterial infection could, in fact, have a direct contribution to the inflammatory immune response.

**Figure 2:**
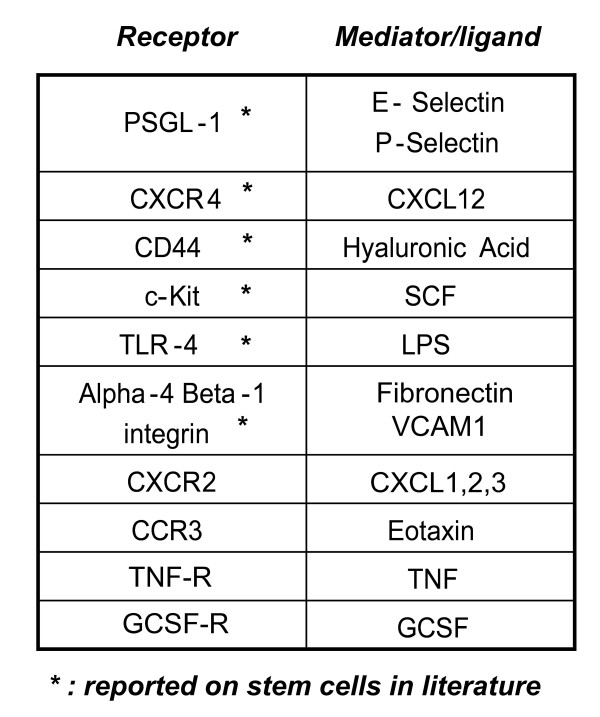
**Inflammatory receptors and mediators shared between stem cells and inflammatory cells**. List of inflammatory receptors and mediators shared between stem cells and inflammatory cells. Modulation of these mediators/receptors was reported to mediate stem cell mobilising, which could allow them to contribute to development of inflammatory and allergic diseases. *: receptors that were reported to be expressed in stem cells.

**Figure 3:**
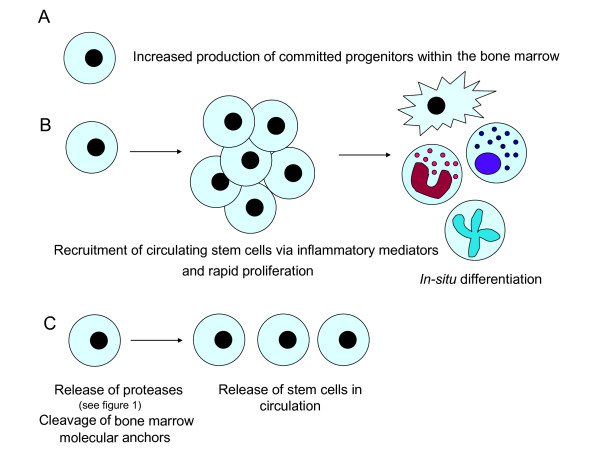
**Proposed models for the role of stem cells in development of inflammation**. **A) **Production of inflammatory mediators during inflammation can increase the number of committed progenitor cells. **B) **Chemotactic mediators released during inflammation can provoke the recruitment of stem cells from the circulation to the site of inflammation where they undergo a rapid proliferation phase, followed by terminal differentiation into inflammatory cells (mast cells, dendritic cells, eosinophils, neutrophils, etc) at the site of the inflammatory reaction, contributing to development and chronicity of disease. **C) **Inflammatory mediators provoke the release of proteases (MMPs) by granulocytes or stem cells within the bone marrow, which cleave the molecular anchors of stem cells and increase their release in the circulation.

In summary, during either steady state trafficking, or in response to potent systemic inflammatory signals, HSCs are well equipped with the appropriate cytokine receptors, chemokine receptors and adhesion and homing molecules for trafficking to the sites of inflammation (see figures 2 and 3). Furthermore, evidence is emerging to suggest that these cells can respond to inflammatory signals with the rapid production of required cell types at the site of inflammation.

### Do stem cells, themselves, influence the further recruitment of inflammatory cells?

#### A role for stem cells in chronic inflammation

Several recent studies have argued that the "stem cells", themselves, could enhance allergy and chronic inflammation via production of inflammatory factors and chemotactic mediators, thereby promoting recruitment of either other precursors or mature hematopoietic cells. Following this line of reasoning, it was shown that human CD34+ progenitor cell supernatants attract RAW 264.7 macrophages cells *in vitro *[30], suggesting that progenitor cell-produced chemotactic agents are released by hematopoietic precursors. Moreover, in a mouse model of angiogenesis, matrigel inserts containing CD34+ progenitor cells were shown to rapidly recruit monocytes/macrophages as well as neutrophils in NOD SCID mice *in vivo*. In this case, it was shown that attraction of monocytes occurred through production of IL-8 and MCP-1 by the CD34+ progenitor cells [31]. An additonal recent study has confirmed that CD34+ human progenitor can act as effector cells in allergy. Indeed, it was shown that they express receptors for the epithelial and mast cells cytokines thymic stromal lymphopoietin (TSLP) and IL-33 [32]. These two cytokines are known to drive towards Th2- IgE dependant allergic reactions. Moreover, when these CD34+ cells were stimulated with the combination of TSLP and IL-33, they produced a variety of cytokines including IL-5, IL-13 and GM-CSF. These three mediators are well known to recruit and promote chronic inflammation in allergy and asthma. Finally, that study demonstrated that the sputum from allergic/challenged patients contained IL-5 and IL-13 positive CD34+ blood cells, confirming that an allergic environment leads to cytokine production by hematopoietic precursors. All in all, this suggests that CD34+ precursors could participate into promoting chronic inflammation.

#### CD34+ cells in mice: a pure population of progenitor cells?

A caveat to this previous model, however, is that CD34 has recently been shown to be expressed on a number of mouse inflammatory cells and precursors (such as mast cells, eosinophils and dendritic cells). Thus, it is possible the inflammatory cytokines detected in this study where produced by more mature CD34+ inflammatory cells rather than CD34+ progenitor cells. Our observation that CD34+ mast cells and their precursors express the same cohort of cell surface markers and antigens[2] suggest that great care must be exercised in distinguishing stem cells from mast cells. Since mast cells are extremely potent elaborators of inflammatory mediators and are present in peripheral tissues at the same frequency as circulating stem cells, this represents a significant problem in the interpretation of many previous studies.

#### Contribution of stem cells to inflammatory disease: a possible clinical implication

With the afore mentioned caveat aside, functional assays have confirmed that bona fide HSCs and hematopoietic precursors, with the ability to generate multiple hematopoietic lineages in lethally irradiated recipients, do circulate through the peripheral blood and lymph and therefore are positioned to home to the sites of inflammation. Although it could be argued that the numbers of these circulating and recruited progenitors would be too low to make a major contribution to allergy and inflammatory disease development, it is important to bear in mind that as HSCs enter the differentiation pathway and become "transient amplifying cells", they exhibit a truly remarkable capacity for proliferation and expansion. This expansion of precursors prior to terminal differentiation and would allow a very limited number of recruited stem cells to have a major impact on local inflammatory cell generation. There are no studies to date that have investigated whether blockade of their recruitment could influence the course of inflammation. However, a closer look at the blockade of some molecules implicated in their trafficking could help us determine whether this avenue could be of therapeutic value.

## Conclusion

The evidence of shared receptor expression and chemotactic potential between inflammatory cells and stem cells is convincing. Moreover, there seems to be a direct effect of inflammation on stem cell recruitment, potentially redirecting the homing of HSCs destined for the bone marrow, to the site of inflammation in diseases like allergy. As the understanding of these interactions grows, we should have a better impression of the extent of stem cell recruitment to the development of allergic inflammatory diseases.

## Competing interests

The authors declare that they have no competing interests.

## Authors' contributions

M-RB and KMM contributed equally to the writing of this manuscript.
